# The complete mitochondrial genome of the freshwater crab *Sinolapotamon patellifer* (Decapoda: Brachyura: Potamoidea)

**DOI:** 10.1080/23802359.2019.1637791

**Published:** 2019-07-12

**Authors:** Wen-Bin Ji, Ying-Yi Cui, Shu-Xin Xu, Xin-Nan Jia, Chun-Chao Zhu, Xian-Min Zhou, Jie-Xin Zou

**Affiliations:** a Research lab of Freshwater Crustacean Decapoda and Paragonimus, School of Basic Medical Sciences, Nanchang University, Nanchang, PR China;; b Department of Parasitology, School of Basic Medical Sciences, Nanchang University, Nanchang, PR China;; c Key Laboratory of Poyang Lake Environment and Resource Utilization, Ministry of Education, Nanchang University, Nanchang, PR China

**Keywords:** Brachyuran, complete mitochondrial genome phylogenetic, *Sinolapotamon patellifer*

## Abstract

We report the complete mitochondrial genome of *Sinolapotamon patellifer* for the first time, which is found to be 16,547 base pairs in length, and contains 13 protein-coding genes (PCGs), two ribosomal RNA (rRNA) genes, 22 transfer RNA (tRNA), and one non-coding AT-rich region known as the D-loop. In addition, the mitogenome has 17 intergenic regions ranging from 1 to 1512 bp in length. The mitochondrial genome of *S. patellifer* is the first mitochondrial genome under the genus *Sinolapamon*, providing DNA data for species identification, enriching the species diversity of Brachyura, and providing a basis for further studies on population genetics and phylogenetics.

The genus *Sinolapotamon* (Tai 1975) (Crustacea: Malacostraca:Decapoda: Brachyura: Potamidae) includes *Sinolapotamon patellifer* (Wu 1934), *Sinolapotamon auriculatum* (Naruse et al. 2010), and *Sinolapotamon palmatum* (Naruse et al. 2010), distributing in mainland China. As a kind of freshwater crab, *S. patellifer* has no genetic data in NCBI. In order to supplement the vacancy of the gene data of *S. patellifer*, we report the complete mitochondrial genome for the first time in this paper. The complete mitochondrial genome of *S. patellifer* will help the identification of species and provide data for further studies on population genetics and phylogenetics.

A male adult specimen of *S. patellifer* was collected from Chayan Village, Jinbao Township, Yangshuo County, Guilin City, Guangxi Province, China in 2017 (N24°46′49.44″, E110°17′34.08″). The sample has been deposited in the Laboratory Specimen Library of Freshwater Crustacean Decapoda and Paragonimus, School of Basic Medical Sciences, Nanchang University, Nanchang, Jiangxi, PR China and National Parasite Germplasm Resources Specimen Library of China with a catalogue number of JX20174073. The sample was fixed in ethanol and then stored at −20 °C before sequence analyses. Genomic DNA extraction, sequencing, gene annotation, and phylogenetic analyses were performed according to the method described by Plazzi et al. (2013). The Bayesian Inference (BI) method was performed using MrBayes vers. 3.2 (Ronquist et al. 2012), with best model GTR + I + G selected using jModelTest vers. 2.1.7. The maximum-likelihood (ML) method was performed using MEGA 6 (Tamura et al. 2013).

There are 16,547 base pairs (bp) in the complete mitogenome of *S. patellifer* (GenBank accession no. MK883709). The genome is rich in AT (76.37% AT content). All genes of the standard metazoan mitogenome are found, including 13 protein-coding genes (PCGs), two ribosomal RNA (rRNA) genes, 22 transfer RNA (tRNA), and one non-coding AT-rich region known as the D-loop. Among those gens, 23 genes are encoded by the Heavy chain (H chain) and 14 genes are encoded by the Light chain (L chain). The total length of the coding genes of *S. patellifer* is 14,755 bp, and the total length of the non-coding region is 1729 bp. There are 17 non-coding regions ranging from 1 to 1512 bp in length. The longest non-coding region locates between *12S rRNA* and *tRNA^Ile^*. There are eight overlapping regions, with a total length of 22 bp and the length ranging from 1 to 7 bp in length. The longest gene overlap region is located between *ND4* and *ND4L*.

The complete mitogenome of *S. patellifer* contains 13 PCGs. Among them, *ND1*, *ND4*, *ND4L*, and *ND5* are encoded in the L chain, and the remaining nine encoded protein genes are encoded in the H chain. The initiation codons of *ATP6*, *ND3*, and *ND6* are ATA, and the initiation codons of the rest PCGs are ATG. The termination codons of all other genes are TAA, except *ATP8* (TAG), *CYTB* (T), and *ND5* (T). The average A + T content of the PCGs is 75.18%. *ND6* has the highest A + T content of 80.64%, and COX1 has the lowest A + T content of 68.75%.

The lengths of 22 tRNA genes of *S. patellifer* are between 61 bp (*tRNA^Cys^*) and 72 bp (*tRNA^Val^*). Fourteen tRNA genes are encoded on the H chain and the rest is encoded on the L chain. All tRNAs except *tRNA^Ser (AGN)^* exhibited a typical clover structure. The *16S rRNA* gene and the *12S rRNA* gene are encoded in the L chain and are 1300 bp and 896 bp in length, respectively. The *16S rRNA* gene is located between *tRNA^Leu (UUR)^* and *tRNA^Va^* while the *12S rRNA* is located between *tRNA^Gln^* and *tRNA^Ile^*.

The longest non-coding region of the mitochondrial genome of *S. patellifer* is located between *12S rRNA* and *tRNA^Ile^*, with a length of 1512 bp and an AT content of 84.79%.

The phylogenetic position of *S. patellifer* in mitogenome relative to other Brachyuran mitogenomes is determined by applying the BI and ML methods on 13 PCGs (Figure 1). Our results indicate that as one of the freshwater crabs, *S. patellifer* is the sister group with *Huananpotamon lichuanense*. This result is consistent with morphological classification and other molecular analyses (Dai 1999; Jia et al. 2018).

**Figure 1.**
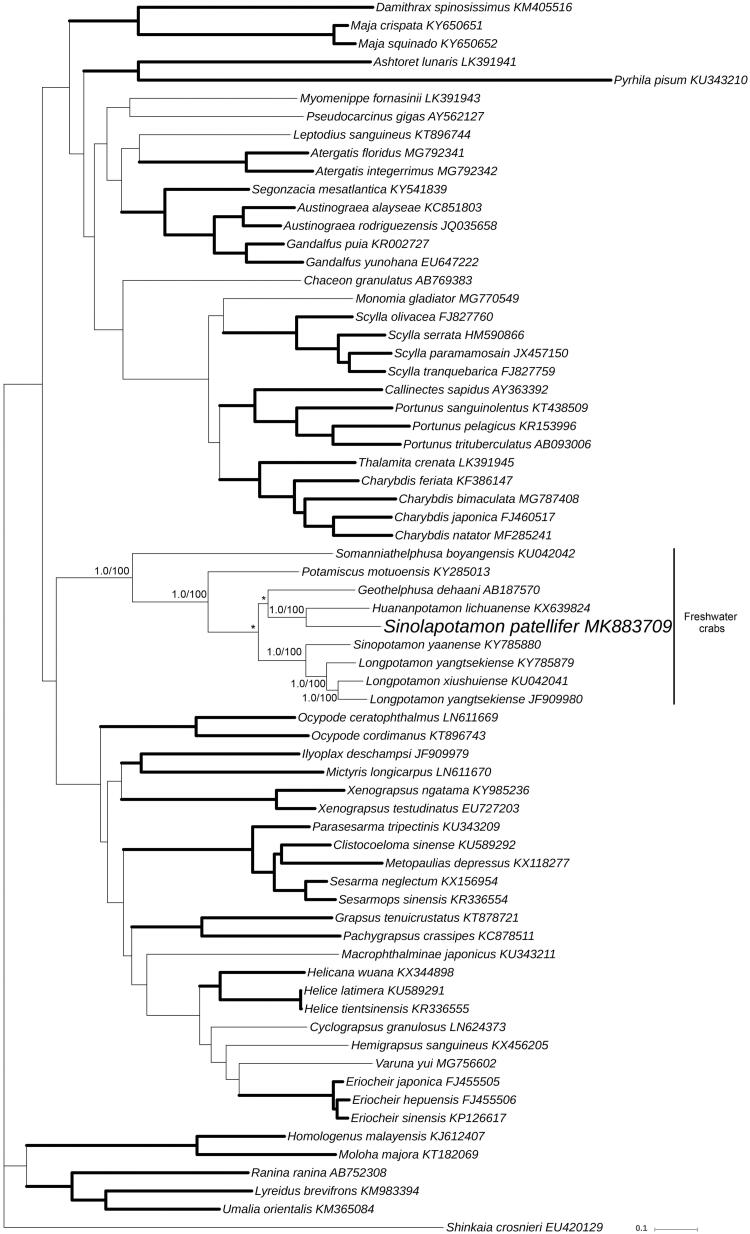
Bayesian Inference (BI) phylogenetic tree of *Sinolapotamon patellifer* and other related brachyurans based on 13 PCGs in mitogenomes. *Shinkaia crosnieri* is served as an outgroup. Numbers on internodes are BI bootstrap proportions and the ML posterior proportions. The same of phylogenetic trees between ML and BI are indicated by bold branches. The differences of freshwater crabs between the ML and BI trees are indicated by ‘*’. The scale bars represent genetic distance.
